# Basal forebrain activation controls contrast sensitivity in primary visual cortex

**DOI:** 10.1186/1471-2202-14-55

**Published:** 2013-05-16

**Authors:** Anwesha Bhattacharyya, Julia Veit, Robert Kretz, Igor Bondar, Gregor Rainer

**Affiliations:** 1 Department of Medicine, University of Fribourg, Chemin du Musée 5, Fribourg 1700, Switzerland; 2 Center for Cognition, University of Fribourg, Chemin du Musée 5, Fribourg 1700, Switzerland; 3 Institute of Higher Nervous Activity & Neurophysiology, Russian Academy of Sciences, Butlerova Str. 5a, Moscow 117485, Russia

**Keywords:** Gamma oscillations, Orientation tuning, Cholinergic

## Abstract

**Background:**

The basal forebrain (BF) regulates cortical activity by the action of cholinergic projections to the cortex. At the same time, it also sends substantial GABAergic projections to both cortex and thalamus, whose functional role has received far less attention. We used deep brain stimulation (DBS) in the BF, which is thought to activate both types of projections, to investigate the impact of BF activation on V1 neural activity.

**Results:**

BF stimulation robustly increased V1 single and multi-unit activity, led to moderate decreases in orientation selectivity and a remarkable increase in contrast sensitivity as demonstrated by a reduced semi-saturation contrast. The spontaneous V1 local field potential often exhibited spectral peaks centered at 40 and 70 Hz as well as reliably showed a broad γ-band (30-90 Hz) increase following BF stimulation, whereas effects in a low frequency band (1-10 Hz) were less consistent. The broad γ-band, rather than low frequency activity or spectral peaks was the best predictor of both the firing rate increase and contrast sensitivity increase of V1 unit activity.

**Conclusions:**

We conclude that BF activation has a strong influence on contrast sensitivity in V1. We suggest that, in addition to cholinergic modulation, the BF GABAergic projections play a crucial role in the impact of BF DBS on cortical activity.

## Background

Cholinergic neuromodulation is mediated by several basal forebrain (BF) structures including the nucleus basalis of Meynert (NBM), which send cholinergic projections to the cortex [1,2]. These cholinergic projections play an important role in various cognitive functions including learning, memory formation and attention [3-5]. Behaviorally, immunotoxic lesions of the cholinergic system have profound effects on learning [6-9], consistent with a large body of evidence linking the blockade of the muscarinic acetylcholine receptors (mAChR), as well as to a lesser extent the nicotinic acetylcholine receptors (nAChR), to impairments in memory formation as well as stimulus discrimination and attention [10,11]. Behavioral performance with novel stimuli appears to be particularly affected by mAChR blockade [12,13]. On the other hand, application of cholinergic agonists can have beneficial effects on behavioral performance. For example, nAChR agonist application enhances the detection performance for low contrast stimuli [14] and administration of the Acetylcholine-esterase inhibitor physostigmine enhances attentional performance [15,16]. These behavioral effects are thought to be mediated by the impact of cholinergic neuromodulation on cortical information processing. Thus, pairing cholinergic activation with sensory stimulation boosts subsequent responses to sensory stimuli in both auditory and visual cortex [17-19] as well as promoting cortical map plasticity [20,21]. Consistent with this, it has been shown that mAChRs play an important role in synaptic plasticity [22-24].

Effects of cholinergic neuromodulation on sensory processing have been extensively investigated using iontophoretic drug application, with much effort having been directed at studies of the primary visual cortex (V1). Early studies have described response increases following the application of cholinergic agonists [25-27] and more recent work has begun to link particular aspects of cholinergic neuromodulation to specific receptor types and cortical laminae [28-31]. By comparison to this pharmacological work, there are relatively few studies examining how cortical processing is affected by electrical BF stimulation, which evokes endogenous Acetylcholine (ACh) release in the cortex [32-34]. This is somewhat surprising given that BF stimulation is currently being tested for clinical use in patients suffering from brain disorders linked to cholinergic dysfunction such as Alzheimer’s disease and Lewy body dementia [35,36]. We therefore aimed to investigate the effects of BF stimulation on sensory processing in the visual cortex, and link the observed effects to our previous results of selective nAChR and mAChR stimulation [37]. An important pertinent aspect is that the BF also sends substantial GABAergic projections to both cortex and thalamic structures [38,39], providing additional routes by which the BF can exert an influence on cortical processing. In our analysis of BF stimulation effects, we focused on spectral changes in the V1 local field potential (LFP) in the absence of visual stimulation, and on the contrast sensitivity [40,41] and orientation selectivity [42,43] of V1 single (SUA) and multi-unit activity (MUA) in response to drifting grating stimuli.

## Results

**Figure 1:**
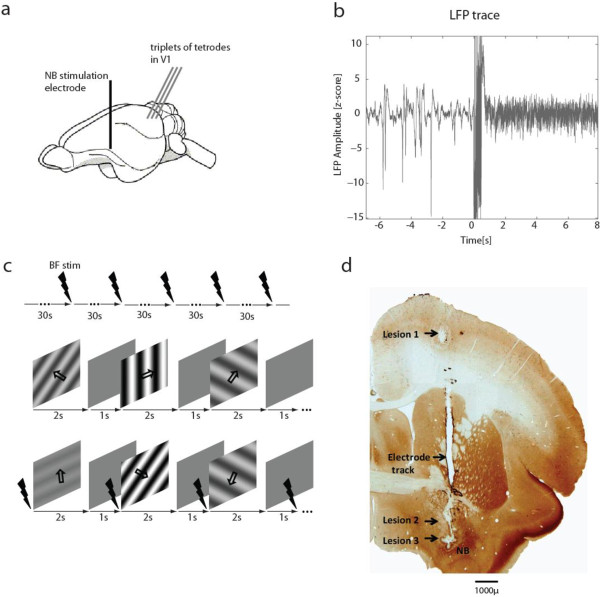
**Experimental design and procedure. **(**a**) Schematic representation of the lateral view of a tree shrew brain showing the position of the stimulation electrode in the basal forebrain (BF) and the three tetrodes used for recording from primary visual cortex (V1). (**b**) Sample time course showing the effect of BF stimulation on spontaneous V1 LFP. Large amplitude, slow fluctuations change to a higher frequency oscillation. (**c**) Experimental Paradigm: The upper panel represents the “BF stim only” protocol used for measuring the effects of BF stimulation on the spontaneous LFP spectrum. The middle and lower panel represent the “BF stim grating” protocols where drifting sinusoidal gratings of different contrasts and orientations were presented, without (middle) and with interleaved BF stimulation (lower panel). The time point of BF stimulation is represented by a black arrow. (**d**) A cytochrome oxidase stained coronal section showing the electrode track and electrolytic lesions at 3 different depths (8200, 7200 and 1000 μm). The lowest lesion, in the region of NBM, was at the final position of the stimulation electrode.

We studied the effects of basal forebrain (BF) microstimulation on neural responses in tree shrew V1 recorded using pairs or triplets of tetrodes (see Figure 1a). During each experiment, we first placed the tetrodes in the visual cortex and determined the approximate receptive field locations for multi-unit activity (MUA) on each tetrode. We then advanced the BF stimulation electrode on a vertical track towards the depth corresponding to the NBM, until stimulation triggered an increase in the γ-band (30-90 Hz) power of the V1 LFP. An example V1 LFP time course demonstrating this spectral change is shown in Figure 1b. Keeping the BF stimulation electrode at this location, we proceeded to record neural activity at different cortical depths in V1. For each V1 location, we first estimated the LFP power spectral density (PSD) change triggered by BF stimulation in the absence of a visual stimulus. We then recorded spiking and LFP activity in V1 in response to drifting grating stimuli of varying contrast and orientation for visual stimulation alone and interleaved visual and BF stimulation (see Figure 1c). At the end of the recording sessions, we made multiple electrolytic lesions along the BF stimulation electrode track, and verified their position using cytochrome oxidase immunohistochemistry. An example is shown in Figure 1d with three lesions, the lowest of which corresponded to the BF stimulation site located in the medial part of the NBM.

**Figure 2:**
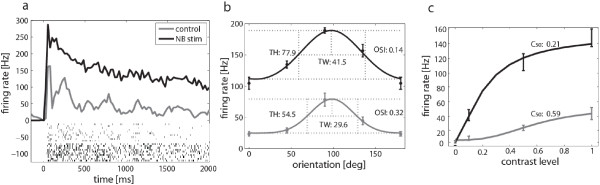
**Example unit, showing increased orientation tuning and contrast sensitivity following BF stimulation.** (**a**) Peri-stimulus time histogram (25 ms binning) and raster plot, showing that BF stimulation led to a robust increase in firing rate (**b**) A wrapped Gaussian fit to the mean firing rate for each orientation is shown. Values of the tuning width (TW) and tuning height (TH), as well as the orientation selectivity index (OSI) are shown next to the corresponding curve. BF stimulation increased TH and TW parameters while decreasing the OSI. (**c**) A Naka-Rushton function was fit to the contrast response data to determine the semi saturation contrast (C_50_ ) as a measure of contrast sensitivity. BF stimulation decreased the C_50_ and thus enhanced contrast sensitivity.

We first examined the effects of BF stimulation on visually evoked activity elicited by drifting grating stimuli that we presented at three contrast levels (10%, 50% and 100%) and eight drift directions corresponding to four orientations. We studied multi-unit activity (MUA) at 87 sites located at different V1 cortical depths. For most sites, MUA varied significantly (3-way ANOVA, P<0.05) as a function of stimulus contrast (86/87 sites) and BF stimulation (83/87), and about 1/3 of the sites exhibited orientation selectivity (31/87). Significant interactions were most common for contrast/BF stimulation (15/87), with only very few sites (<4/87) showing other 2-way or 3-way interactions. A representative MUA response, averaged across contrasts and orientations, is shown in Figure 2a, demonstrating an approximately 4-fold elevation of activity from 26 to 101 Hz following BF stimulation. Similarly, across the population there was an average 2.7 fold elevation of activity from 29.6 to 80.0Hz following BF stimulation (P<<0.001), with 84 units showing activity increases and only 3 showing reductions. The orientation tuning function at 100% contrast for this example unit is shown in Figure 2b. In addition to generally elevating firing rate, BF stimulation resulted in an increased tuning height (TH), and a reduction in orientation tuning as evidenced by a lower orientation selectivity index (OSI) and increased tuning width (TW). For the same unit, the contrast response function (CRF) averaged across orientations is shown in Figure 2c. Fitting a Naka-Rushton function to these data, we found a robust elevation in baseline-subtracted peak firing rate (R_max_) whereas baseline firing rate (R_0_) was unaffected. Contrast sensitivity was measured by the semi-saturation contrast (C_50_) parameter that takes small values for units that exhibit robust activity already at relatively low contrast values. For the example unit, we observed reduced C_50_ values and thus increase in contrast sensitivity following BF stimulation.

**Figure 3:**
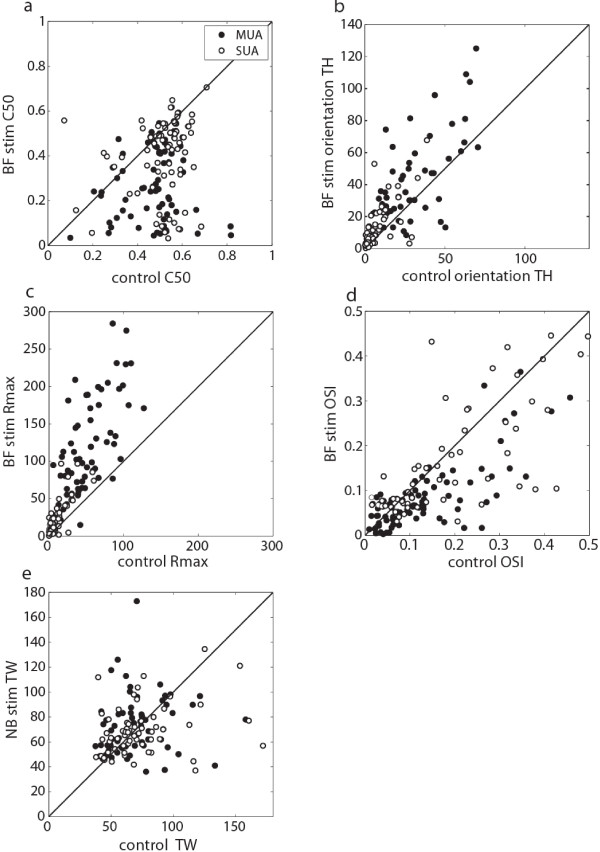
**Population data for BF stimulation effects on orientation and contrast parameters of MUA and SUA.** MUA sites are plotted as filled circles, SUA as open circles (**a**) contrast sensitivity is increased by BF stim (decreased C_50_ values) (**b**) values for both tuning height (TH) and (**c**) baseline-subtracted peak firing rate (R_max_) are increased (**d**) the Orientation selectivity index (OSI) is significantly decreased while (**e**) there is no systematic effect on tuning width (TW).

We examined orientation tuning and contrast response parameters for the population of 87 MUA sites, as well as for a population of 84 isolated single neurons (see methods). We observed a marked decrease in the contrast sensitivity C_50_ parameter for both MUA and SUA, as shown in Figure 3a (control/BFstim MUA: 0.49 / 0.31, SUA: 0.51 / 0.40, P<<0.01). Amplitudes of orientation tuning and contrast response functions were increased for both MUA and SUA (see Figure 3b, c), as demonstrated by increases in TH (control/BFstim MUA: 25.0 Hz / 36.1 Hz, SUA: 7.4 Hz / 12.6 Hz, P<<0.01) and R_max_ (control/BFstim MUA: 42.1 Hz / 96.7 Hz, SUA: 8.7 Hz / 18.7 Hz, P<<0.01) parameters. On the other hand, OSI values were decreased (control/BFstim MUA: 0.13 / 0.09, P<<0.01, SUA: 0.30 / 0.26, P<0.05) as shown in Figure 3d, while TW parameters remained unchanged (control/BFstim MUA: 68.7° / 72.1°, SUA: 74.3° / 69.5°, P>0.1, see Figure 3e). Decreased OSI values despite unchanged TW and increased TH can be explained by the upward shift of the orientation tuning function following BF stimulation, since the OSI is invariant to multiplicative but not additive firing rate changes. None of the five parameters exhibited pronounced variations across cortical layers, as demonstrated by a lack of significant interactions between layer (supragranular, granular, infragranular) and BF stimulation condition (2-way ANOVA, P>0.1). Overall, BF stimulation had similar effects on V1 MUA and SUA, increasing firing rates, while decreasing orientation selectivity and leading to a pronounced increase in contrast sensitivity.

**Figure 4:**
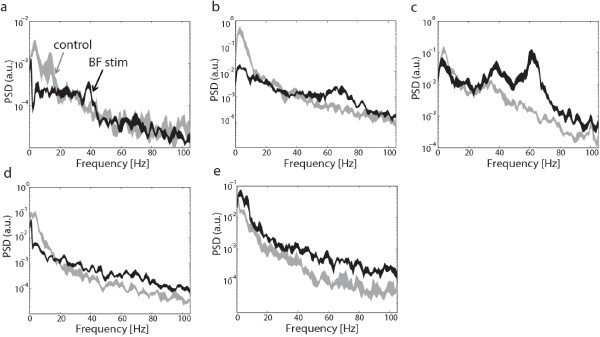
**Examples of V1 LFP power spectral density (PSD) changes triggered by BF stim.** The gray and black traces represent the PSD ± SEM before and after BF stimulation respectively. The different panels represent examples for the different observed patterns: (**a**) 40Hz γ-band peak (**b**) 70Hz γ-band peak (**c**) Both 40Hz and 70Hz γ-band peaks (**d**) Decrease in the low frequency (0–10Hz) band and broad increase in the γ-band. (**e**) Increase in both low and high frequency band, no peaks.

**Figure 5:**
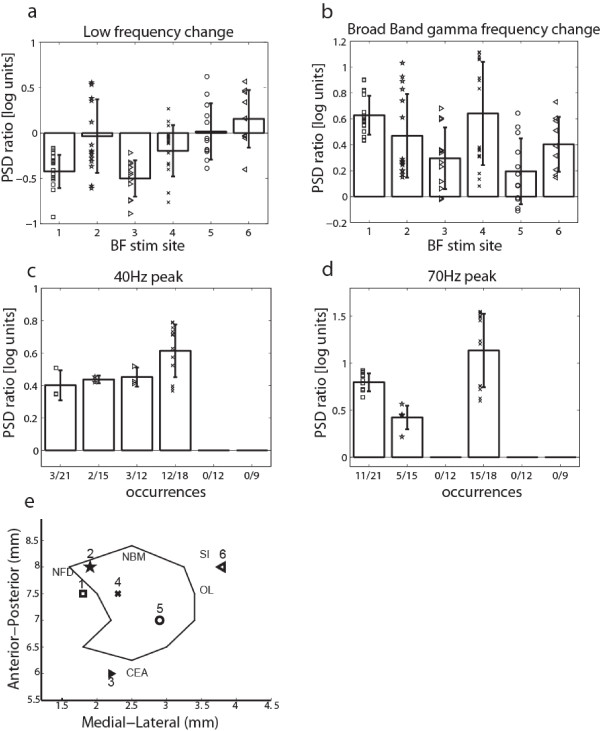
**Spectral V1 LFP effects for different BF stimulation sites.** The bar plots show the average LFP PSD ratio in log units between BF stimulation and control condition, separately for each BF site. Error bars: SD. The different symbols represent spectral changes at multiple V1 recording sites for each respective BF site. (**a**) Low frequency band effects were variable, with 3/6 sites showing significant reductions. (**b**) The broad γ-band showed a significant increase for all BF sites. (**c**) A 40Hz peak was seen in 4/6 BF sites. The number of V1 locations at each site exhibiting the 40Hz peak is shown below each bar plot. (**d**) A 70Hz peak was observed in 3/6 experiments. (**e**) Schematic representation of the BF stimulation sites in a horizontal projection. Each site is represented by a unique symbol and a corresponding number. The black outline shows the approximate boundary of the NBM. Structures adjacent to the NB are marked in their approximate locations: NFD: Nucleus fasciculi diagonalis Brocae; SI: Substantia Inominata; OL: Nucleus tractus olfactorii lateralis; CEA: Nucleus Centralis Amygdalae.

A qualitative evaluation of the V1 LFP spectral changes triggered by BF stimulation revealed a number of different patterns, which are illustrated using some representative examples in Figure 4. Since γ-band increase was used as criterion for stimulation position selection, this spectral feature was present at most sites. We commonly observed low frequency decreases accompanying the γ-band increases (panels a-d), and less frequently a joint elevation of both low and high frequencies, as shown in panel (e). In addition, BF stimulation sometimes elicited peaks in the γ-band that appeared to fall into two categories with center frequencies around 40 Hz (panels a,c) and 70 Hz (panels b,c). For a quantitative analysis of V1 LFP spectral changes, we examined a low frequency band between 1 and 10Hz and a broad γ-band between 30 and 90Hz, as well as manually determined frequency bands corresponding to the 40 Hz and 70 Hz γ-band peaks wherever present. For each frequency band, we compared average LFP PSD for BF stimulation to a control condition recorded immediately prior to stimulation. For the low frequency band we found significant reductions in 3/6 experiments (P<0.05), whereas effects were highly variable in the remaining experiments with both increases as well as decreases (see Figure 5a). As expected from the stimulation electrode placement, PSD averaged across the entire γ-band exhibited significant increases following BF stimulation for all of the experiments (P<0.05), as shown in Figure 5b. Neither low frequency nor broad γ-band changes appeared to vary by layer (one-way ANOVA, P>0.1). Examining the γ-band peaks, we found that 40 Hz and 70 Hz peaks occurred in 4 and 3 experiments and during a total of 20/87 (23%) and 31/87 (36%) stimulation blocks respectively (see Figure 5c, d). To relate the spectral V1 LFP changes to BF anatomy, we plotted the location of the BF stimulation electrode in a horizontal projection for each animal, including positions within the NBM as well as adjacent structures (see Figure 5e). Based on histological verification of each recording site, three stimulation sites fell within the NBM, while the remaining ones were in close proximity [44]; medially in the nucleus fasciculi diagonalis Brocae (NFD), laterally close to substantia inominata (SI) and posteriorly close to nucleus centralis amygdalae (CEA). We note that γ-band peaks occurred only for sites located medially (sites 1–4), encompassing both sites within as well as in close proximity to the NBM. Consistent with this observation, the BF stimulation dependent effects on broad γ-band PSD were negatively correlated with the medial/lateral position of the stimulation electrode (R=−0.25, P<0.05).

**Figure 6:**
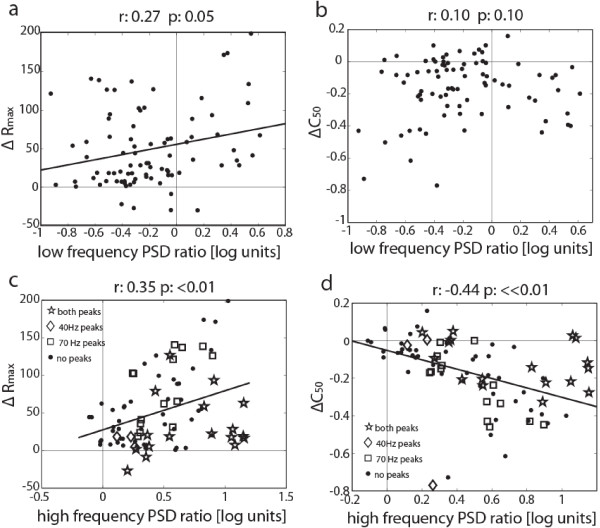
**Relationship between LFP PSD ratios and contrast response changes of V1.** MUA Rmax changes (Δ R_max_) were significantly correlated with both (**a**) low and (**c**) high frequency changes. Semi-saturation contrast changes (ΔC_50_) were (**b**) uncorrelated with low frequency changes but (**d**) showed a strong negative correlation with γ-band changes. The large symbols in (**c**) and (**d**) denote the recording locations showing either a 70 Hz peak (square), a 40 Hz peak (diamond), both peaks (star) or no peaks (dot). Correlation coefficients (r) and p-values are given on top of each plot.

Finally, we wanted to relate the V1 LFP spectral changes to BF stimulation in the absence of visual input to contrast and orientation tuning parameters. We found no significant correlations between low or high frequency LFP spectral changes and BF stimulation related changes of any orientation related parameters TW, TH and OSI (Pearson correlations, P>0.09). For contrast response function related parameters, we did find a number of significant correlations: Low frequency PSD ratios were correlated with ΔR_max_ (r=0.27, P<0.05), but not C_50_ (r=0.10, P>0.1) changes following BF stimulation, as shown in Figure 6a,b. Interestingly, the correlation between ΔR_max_ and low frequency PSD ratio was mainly due to the cases where robust increases in low frequency PSD were seen following BF stimulation, and the correlation was no longer significant if these cases were removed from the population (r=−0.14, P>0.1). PSD ratios in the high frequency band were robustly correlated with both ΔR_max_ (r=0.35, P<0.01) and ΔC_50_ (r=−0.44, P<<0.01), as shown in Figure 6c, d. There was no apparent difference in the R_max_ and C_50_ distributions between the sites that exhibited LFP spectral peaks at 40 and/or 70Hz and those sites that did not. In addition, considering only sites that showed a 40 or 70Hz peak, we found no correlation between the BF stimulation-dependent peak magnitude change and ΔR_max_ (r_40Hz_=0.08 P=0.8, r_70Hz_ =−0.18 P=0.3) or ΔC_50_ (r_40Hz_ =−0.07 P=0.8, r_70Hz_ =0.04 P=0.8). Thus, BF stimulation at sites that elicited a spectral LFP peak did not generally produce stronger R_max_ or C_50_ changes than sites with a broad γ-band increase. Taken together, the magnitude of the γ-band PSD change in the absence of visual input was the best predictor for contrast sensitivity increases in V1 MUA, while being largely unrelated to V1 orientation selectivity.

## Discussion

We investigated how BF stimulation affected sensory representations by examining parameters related to the contrast response and orientation selectivity of V1 units. We observed strong increases in firing rates, a pronounced increase in contrast sensitivity and decreases in orientation selectivity.

Since the BF, where we stimulated in the present study, contains a large population of cholinergic cortical projection neurons [2,45], the results of BF stimulation thus might be thought to have been predictable based on the results of pharmacological application of ACh or specific agonists of nAChR and mAChR types in V1. Iontophoretic ACh application has both excitatory and inhibitory effects on unit activity, with most studies reporting about twice as many increased compared to suppressed units [25,27,31,46,47], but see [29]. Selective stimulation of the nAChR has been shown to elicit facilitation in a great majority of units, particularly in the granular input layer of cortex [28,37]. Similarly, mAChR stimulation also tends to elicit facilitation, although these effects appear to occur in all cortical layers [27,31,46]. Comparing our present findings to our previous data on iontophoretic cholinergic drug application in tree shrew V1 and taking the change in baseline-subtracted peak firing rate (ΔR_max_) as a measure of effect size for facilitation, we observed average values of +25%, +45% and +130% for mAChR, nAChR and BF stimulation respectively. The ΔR_max_ value following BF stimulation is thus greater than the sum of the increases due to the two cholinergic receptor types, although release profiles following iontophoretic drug application and BF stimulation may differ, complicating direct comparisons. Nevertheless, it seems that cholinergic BF projections to the cortex are unlikely to be the only mechanism involved in the increase of cortical firing rates. A stronger case for non-cholinergic contributions following BF stimulation can be made concerning contrast sensitivity changes. To our knowledge, our findings in fact represent the first demonstration of a physiological intervention that reduces C_50_ values in the visual cortex and thus enhances the ability of neurons to detect low contrast stimuli. Accordingly, C_50_ values are unaffected by ACh application [29,31] nAChR [28,37] or mAChR stimulation [31,37], GABA-A blockade [48] or alteration of parvalbumin-containing GABAergic interneuron activity [49]. Furthermore, it has been shown that C_50_ values in the LGN are unaffected by both alertness levels and isoflurane anesthesia [50,51]. The lack of V1 C_50_ modulations in response to cholinergic pharmacological agents suggests that reductions in C_50_ values in response to BF stimulation cannot be accounted for by BF cholinergic projections alone.

**Figure 7:**
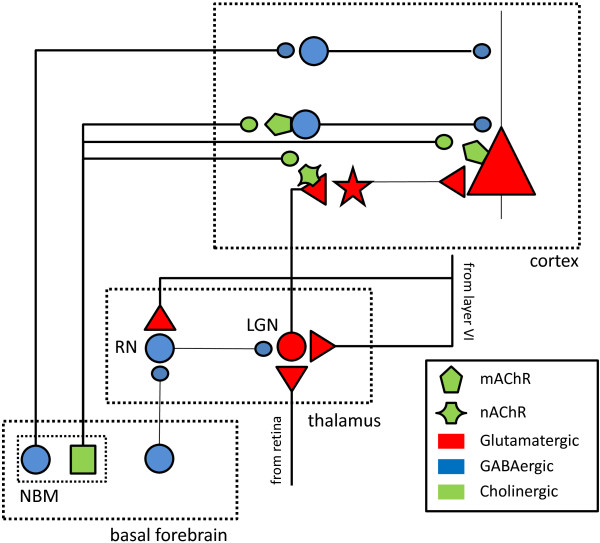
**Simplified representation of BF, cortical and thalamic circuitry illustrating possible mechanisms of observed effects of BF stimulation.** Cholinergic neurons in the NBM can influence cortical activity through either muscarinic receptors (mAChRs) located on both pyramidal neurons as well as inhibitory interneurons or through nicotinic receptors (nAChRs) located mostly presynaptically on the thalamocortical terminals. GABAergic neurons of the NBM also project to the cortex where they preferentially target other GABAergic interneurons. The effect of stimulating these neurons would thus mediate disinhibition in cortex. Another population of GABAergic neurons in the BF innervates the thalamic reticular nucleus, where - by a similar disinhibitory mechanism - they could enhance sensory signals relayed by the LGN from the retina to the cortex. Feedback connections from cortex to the LGN, which may contribute to BF stimulation related regulation of activity, are also shown.

It thus appears difficult to explain both the increased responsiveness of cortical neurons and the increased contrast sensitivity entirely based on BF cholinergic projections to cortex. There are two potential explanations for this finding, which are illustrated in Figure 7, a simplified schematic representation of the projections from the NBM of the BF to the cortex. The main cortical sites of cholinergic action are the mAChRs on pyramidal and GABAergic cells, and the nAChRs on the presynaptic terminals of the thalamo-cortical projection neurons. Activation of mAChRs can thus cause excitation in pyramidal cells directly [52-54], or inhibit pyramidal cell activity by activating parvalbumin-containing GABAergic neurons [46,55-57]. Activation of nAChRs mainly enhances pyramidal cell activity by increasing glutamate release from thalamic terminals [28,37,58]. The GABAergic projections constitute another separate pathway by which BF activity can modulate cortical function [59,60]. These BF GABAergic projections preferentially contact somatostatin/calbindin containing GABAergic cortical interneurons [56], but see [61], and can thus cause elevated activity in pyramidal neurons by disinhibition. We suggest that this disinhibition is one candidate mechanism that might be responsible for the large (ΔR_max_ =130%) increases in activity we observed to BF stimulation in comparison to iontophoretic cholinergic receptor activation. During BF stimulation, the GABAergic BF neurons reduce activity in some cortical GABAergic interneurons, whereas activity of other GABAergic cortical interneurons is increased by cholinergic BF neurons via mAChR stimulation. Consistent with this hypothesis, application of a GABA-A antagonist in cortex, which blocks inhibition on pyramidal neurons by both of the inhibitory pathways mentioned above, results in even stronger elevation of activity (ΔR_max_ =240%) [48]. The reduced C_50_ value could also be due to disinhibition of cortical pyramidal neurons by this mechanism, suggesting that contrast sensitivity is controlled by neural circuits involving somatostatin/calbindin cortical interneurons, rather than parvalbumin containing interneurons or GABAergic circuits in general [48,49,56]. Consistent with this hypothesis, a recent study has shown that optogenetic activation of V1 somatostatin neurons in fact significantly reduces V1 contrast sensitivity, thus leading to increased C_50_ values [62]. The second candidate mechanism involves the disinhibition of thalamic relay cells via projections of the BF to the reticular nucleus [39,63]. This is consistent with recent observations that BF stimulation increases activity of thalamic relay cells in the LGN [33]. An elevation in trial-to-trial reliability of neural responses was also observed in that study, which we suggest might have been a result of increased contrast sensitivity. After BF stimulation, units will respond to low contrast visual features of the movie stimulus that they were unresponsive to before, as well as responding more consistently across trials to intermediate contrast visual features. Note that the effects on the LGN associated with BF stimulation may also be due to cortico-thalamic feedback from layer VI of cortex [64,65].

Cholinergic activation has been shown to have relatively weak effects on selectivity to stimulus features such as orientation and direction in V1, with studies reporting moderate decreases [27,46,47] as well as increases [25,26]. Our own previous results in tree shrew V1 have suggested slight increases and decreases of orientation selectivity mediated by mAChRs and nAChRs respectively [37]. Generally consistent with previous findings, we report here unchanged TW, increased TH and decreased OSI values in response to BF stimulation. The relatively large decrease in OSI values – despite the increase in tuning height and unchanged width – is a result of the large general increase in V1 firing rates, even for non-preferred orientations.

Increase in broad γ-band activity in the cortex has been linked to BF activation [66-70], and – in addition to reductions in low frequency activity – is used as a relevant aspect of the cortical spectrogram for the placement of BF stimulation electrodes [33,67]. Here, we found that γ-band effects could take the form of broad-band increases, as well as displaying peaks with center frequencies around 40 Hz and 70 Hz. Notably, dual peaks with similar center frequencies have been previously observed in visual cortical slice preparations during mAChR stimulation [71,72]. Similarly, it has been shown that γ-band oscillations with a somewhat lower peak frequency of 26 Hz can be evoked in the visual cortex *in vivo* by application of the cholinergic agonist Carbachol [73]. This suggests that the γ-band peaks we observed in visual cortex following BF stimulation are likely to be a result of cholinergic BF projections to the cortex that target mAChRs, which in turn up-regulate perisomatic GABAergic inhibition. Interestingly, we were able to elicit γ-band peaks only at medial BF sites within and close to the NB, whereas stimulation at more lateral BF sites did not elicit any peaks while nevertheless evoking increased broad γ-band activity. We speculate that medial stimulation sites might thus be more suitable for activating BF cholinergic projections to the cortex, possibly by targeting fibers of passage that initially take a medial course from the NBM, before projecting posteriorly [74]. Our correlation analyses suggest that the γ-band peaks do not predict firing rate (ΔR_max_) or contrast sensitivity (ΔC_50_) increases following BF stimulation. Instead, it is the broad γ-band activity which is correlated with both ΔR_max_ and ΔC_50_ values, suggesting that the overall strength of γ-power, rather than the appearance of specific peaks is related to the main effects on V1 unit activity. Generally, γ-oscillations are thought to be generated by the interplay between local excitatory and inhibitory coupled networks [75]. It is therefore likely that, in addition to the cholinergic BF projections to the cortex, the two GABAergic pathways originating from the BF (see Figure 7) also contribute to the increase of γ-band activity. This could be accomplished by shifting the balance between excitation and inhibition in cortex through an up-regulation of thalamo-cortical excitatory drive and the reduction of GABAergic inhibition onto cortical pyramidal cells, through the BF GABAergic projections to the reticular nucleus and cortex respectively. An involvement of GABAergic cortical projection pathways is consistent with modeling work suggesting that reduced drive to a set of cortical interneurons leads to γ-oscillations in a coupled network of excitatory and inhibitory neurons [76]. At the same time, the GABAergic projection to the reticular nucleus could also play a role, consistent with the recent demonstration that coupled inhibitory networks in conjunction with long range excitation can generate broad γ-band activity without ostensible spectral peaks [77].

## Conclusion

In summary, our major finding is a strong increase in the contrast sensitivity of V1 neurons as well as a large increase in neural responsiveness following BF DBS. Converging evidence suggests that these effects are unlikely to be due to the action of cholinergic mechanisms alone. We suggest that the action of GABAergic BF projection pathways is a candidate mechanism that could account for the observed findings, by causing disinhibition of V1 pyramidal neurons. This disinhibition may also contribute to the reduced stimulus selectivity we observed following BF stimulation, consistent with previous findings showing reduced stimulus selectivity in V1 as well as inferior temporal cortex following GABA receptor blockade [48,78]. Given that these effects are likely to be detrimental for visual discrimination performance [79], it is important to carefully consider the co-activation of GABAergic, in addition to cholinergic BF projections in clinical applications of BF stimulation.

## Methods

### Ethical approval

All experiments were approved by the “Tierversuchskommission des Kantons Fribourg” and were in full compliance with applicable Swiss as well as European Union directives.

### Animal preparation

Experiments were performed on six adult tree shrews (*Tupaia belangeri*) aged 3–9 years. Animals were prepared as described previously [80]. Briefly, experiments were carried out in anesthetized and paralyzed animals (0.5%-1.5% Isoflurane in Oxycarbon (95%O_2_, 5%CO_2_), Pancuroniumbromide i.p.) that were artificially respirated at 100 strokes per minute.

### Electrophysiology

Stimulation electrodes consisted of two Teflon insulated Platinum/Iridium Pt90/Ir10 wires (0.05mm diameter), twisted together and placed inside a Silica guide tube (250 μm ID, 350 μm OD). Impedances ranged from 500kΩ to 1.2MΩ. The stimulation stereotrode was advanced vertically downwards at the AP-ML coordinates of the NBM using a hydraulic microdrive until a depth of about 7000 μm was reached. We electrically stimulated at this position and observed the changes in the local field potential. If the V1 LFP did not show the expected frequency changes – particularly a γ-band increase – after stimulation, we advanced the stimulation electrode in approximately 200 μm steps further until we observed the expected V1 LFP spectral signature, at which point we left the stimulation stereotrode in place for the rest of the experiment. We then recorded neural activity from different depths in V1, conducting at each site first a “BF stim only” protocol to quantify the spectral changes in the spontaneous LFP, and then a “BF stim grating” protocol with and without BF DBS in separate blocks.

Tetrodes were fabricated by twisting together four 12.7 μm-diameter nickel–chromium wires (RO-800; Kanthal Precision Technology) and the impedances were reduced to 200–300 kΩ by gold plating. Two or three tetrodes were advanced into the primary visual cortex using a manual microdrive (David Kopf Instruments). Similar penetrations were made in all experiments close to normal, to the cortical surface by tilting the micro drive back at an angle of approximately 30°. For a given penetration, we recorded activity at multiple depths typically spaced around 200 μm apart. The signal was amplified by a RA16PA Medusa preamplifier and then filtered and digitized by a RZ5 Bioamp Processor (Tucker-Davis Technologies, Alachua, FL). LFPs were filtered between 1 and 200 Hz and sampled at 509 Hz. To estimate multi-unit spiking activity (MUA), we thresholded signals that were filtered between 300 Hz and 4 kHz and sampled at 24.4 kHz, on each tetrode by using the channel with largest signal to noise ratio. We focus on MUA because a major goal of the study was to relate BF stimulation related LFP spectral changes to effects on spiking activity at various V1 sites, requiring a single spiking activity related signal for each V1 recording site, as well as for direct comparability to our previous pharmacological work [37]. For a subset of analyses related to orientation selectivity and contrast sensitivity, we additionally examined results for single unit activity (SUA) for a population of well-isolated neurons (n=84). SUA was isolated using manual tetrode clustering software (MClust, http://umn.edu/~redish/mclust).

### Histology

At the end of a recording session, we made reference lesions at multiple depths both in the visual cortex and along the penetration to the NBM using a constant current stimulator (WPI A360). Lesions were made by passing 10 μA for 10s in V1, and 250 μA for 30 or 60s in the BF. Animals were then perfused through the heart with 0.9% NaCl followed by ice cold 4% PFA in 0.1M phosphate buffer (pH 7.4). The top of the skull was removed, and stereotactic coronal cuts were made through the brain allowing the extraction of brain segments containing V1 and BF respectively. The brain was then removed and immersed in a mixture of 2% DMSO and first 10% and later 20% glycerol in 0.1M phosphate buffer (pH 7.4). The V1 brain segment was cut into 50 μm sagittal sections and the BF segment into 50 μm coronal sections using a freezing microtome (Microm HM440E). Cytochrome oxidase immunohistochemistry was performed on the sections for the localization of the lesions [81]. In V1, recording locations were assigned to layers (supragranular, granular and infragranular) based on anatomical reconstruction of recording positions using histology when possible (n=4 animals), and estimated based on actual recording depth and average borders between layers correcting for the non-perpendicular angle of tetrode penetration (n=2 animals) [80].

### Electrical and visual stimuli

Electrical stimulation pulse trains were generated with a Pulsar 6i (FHC, Bowdoinham, ME 04008, USA) and usually consisted of a 500 ms long train of constant 7 to 10V positive pulses of 50 μs duration at 100Hz delivered through one of the two wires of the stereotrode only (unipolar stimulation; the return path being the ground screw). Visual stimuli were generated with Psychophysics Toolbox running on a Mac Mini and presented on a gamma corrected 21” diameter (56.7° visual angle) Compaq Qvision 210 cathode ray tube monitor running at 119.22 Hz. Maximum luminance measured with a Minolta TV-color analyzer was determined as 50 cd/m^2^. Before recording neural activity, we mapped the approximate location of the receptive fields of the neurons under study by manually sliding bars generated with a simple graphics program back and forth on the monitor.

For this study we used two different stimulation paradigms: In the “BF stim only” protocol we presented a blank screen of intermediate luminance for the entire length of the recording, collecting at least 30 seconds of spontaneous activity and then electrically stimulated the BF five times with 30s inter stimulation interval. The “BF stim grating” protocol consisted of two blocks (a) visual stimulation using drifting gratings in conjunction with BF stimulation and (b) visual stimulation alone as a control. Drifting sinusoidal gratings were presented at a fixed, manually determined optimal speed (1–3 cycles per second) and spatial frequency (0.03 - 0.7 cycles per visual degree), were chosen to be large enough to cover all simultaneously recorded receptive fields and ranged from 10 to 30 degrees. We showed three different contrast conditions (between 10 and 100%) and eight drift directions spaced uniformly at 45° intervals – note that these conditions correspond to 4 different orientations, each drifting in two opposite directions. The set of 24 different stimuli was presented five times in different pseudorandom order. Each stimulus was shown for two seconds with one second inter-stimulus blank period. In the blocks with the interleaved electrical stimulation, we stimulated the BF before every visual stimulus, immediately following the one second inter-stimulus interval.

### Data analysis

For the “BF stim only” protocol we analyzed the power spectral density (PSD) of the LFP activity using the Matlab implementation of Thomsons multitaper method (function: pmtm, nw = 3, nfft = 1024, fs = 1000, yielding a frequency resolution of ~1Hz) in five, non-overlapping, two second windows before the first BF stimulation and the first two-second window immediately after each BF stimulation, taking care not to include any part of the electrical stimulation artifact. This yielded five independent estimates of the PSD before, and five estimates immediately following each BF stimulation. For further analysis we averaged the PSD estimates between 1 and 10 Hz and between 30 and 90 Hz (11 and 62 frequency bins respectively) across the five repetitions. We report the logarithmic ratio of BF stimulation to control PSD as the PSD ratio for both low and high frequency bands. Visual inspection of the PSD spectra revealed that BF stimulation often resulted in spectral peaks in the “BF stim only” condition without visual stimulation. We observed that peaks occurred near center frequencies of 40 Hz and 70 Hz, with both peaks sometimes occurring together. We determined the borders of each apparent spectral peak by visual inspection, and used this information to compute PSD ratios for each peak occurrence as above. For the changes in visual responses after BF stimulation, we compared the firing rates of each unit in response to the drifting grating stimulus during the two second visual stimulation period with and without preceding electrical BF stimulation. Note that the sequence of BF stimulation followed by sensory processing is somewhat artificial, since during task performance BF neurons are activated after sensory processing of incoming stimuli [82,83].

### Orientation preference and contrast response function

Preferred orientation, as well as tuning strength was extracted from the V1 unit responses to the drifting grating stimulus using an orientation selectivity measure that relies on vector summation:

$$\mathrm{OSI}=\frac{\sqrt{{\left(\sum_{i}^{N}R\left(\theta_{i}\right)\sin\left(2\theta_{i}\right)\right)}^{2}+{\left(\sum_{i}^{N}R\left(\theta_{i}\right)\cos\left(2\theta_{i}\right)\right)}^{2}}}{\sum_{i}^{N}R\left(\theta_{i}\right)}$$

The vectors of the responses to the full contrast gratings *R*(*θ*_*i*_) to each orientation *θ*_*i *_are added up in the complex plane and then normalized by the sum of all responses. The OSI takes a value between 0 for untuned and 1 for perfectly tuned responses. OSI values were computed for 72/84 single neurons and 87/87 MUA sites that exhibited sufficient firing rates (>2 Hz) to allow reliable estimates.

We also fitted a wrapped Gaussian function to the responses to measure the tuning width (TW) and tuning height (TH):

$$G\left(\theta \right)=A_{0}+A\sum_{n=-5}^{n=5}\exp\left(\frac{-{\left(\theta -\mu +180n\right)}^{2}}{2\sigma^{2}}\right)$$

Where *μ* is the predicted preferred orientation, *A* the amplitude of the Gaussian and *A*_*0 *_the offset from zero. TH corresponds to*A*, and TW is defined as full width at half height, calculated as $2\sigma \sqrt{2\;\ln\;2}$. We obtained good fits for 60/84 single neurons and 64/87 MUA sites.

For the contrast analyses, we averaged data at each contrast across all drifting directions, so that each data point represents a mean of 5×8 = 40 trials. We fitted Naka-Rushton functions to the contrast response curve: $r\left(c\right)=R_{\max}\frac{C^{n}}{C^{n}+C_{50}^{n}}+R_{0}$, where the parameters baseline-subtracted peak firing rate (R_max_), baseline firing rate (R_0_) and the semi-saturation contrast (C_50_) are obtained. The C_50_ is inversely related to the contrast sensitivity: the smaller the C_50_, the higher the contrast sensitivity. Reported p-values were calculated using a paired t-test as data were normally distributed according to a Kolmogorov-Smirnov test (p<0.05); We obtained good fits for 72/84 single neurons and 84/87 MUA sites.

## Abbreviations

ACh: Acetylcholine; BF: Basal forebrain; CEA: Nucleus centralis amygdalae; CRF: Contrast response function; DBS: Deep brain stimulation; LFP: Local field potential; mAChR: Muscarinic ACh receptor; MUA: Multi unit activity; nAChR: Nicotinic ACh receptor; NBM: Nucleus basalis of Meynert; NFD: Nucleus fasciculi diagonalis Brocae; OSI: Orientation selectivity index; PSD: Power spectral density; SI: Substantia innominata; TH: Tuning height; TW: Tuning width; V1: Primary visual cortex.

## Competing interests

The authors declare that they have no competing interests.

## Authors' contributions

This work was performed in the Visual Cognition laboratory at the University of Fribourg. Conception and design: AB, JV, IB, RK, GR. Collection and interpretation of data: AB, JV, RK, GR. Drafting and revising article: AB, JV, IB, GR. All authors read and approved the final manuscript.
